# Clinical outcome of hepatocellular carcinoma can be predicted by the expression of hepatic progenitor cell markers and serum tumour markers

**DOI:** 10.18632/oncotarget.25074

**Published:** 2018-04-24

**Authors:** Satoshi Seino, Atsunori Tsuchiya, Yusuke Watanabe, Yuzo Kawata, Yuichi Kojima, Shunzo Ikarashi, Hiroyuki Yanai, Koji Nakamura, Daisuke Kumaki, Masaaki Hirano, Kazuhiro Funakoshi, Takashi Aono, Takeshi Sakai, Jun Sakata, Masaaki Takamura, Hirokazu Kawai, Satoshi Yamagiwa, Toshifumi Wakai, Shuji Terai

**Affiliations:** ^1^ Division of Gastroenterology and Hepatology, Graduate School of Medical and Dental Sciences, Niigata University, Chuo-Ku, Niigata 951-8510, Japan; ^2^ Drug Discovery Laboratories, Chiome Bioscience Inc., 907 Nogawa, Miyamae-Ku, Kawasaki-Shi, Kanagawa 216-0001, Japan; ^3^ Division of Gastroenterology and Hepatology, Niigata Prefectural Central Hospital, Joetsu-Shi, Niigata 943-0147, Japan; ^4^ Division of Surgery, Niigata Prefectural Central Hospital, Joetsu-Shi, Niigata 943-0147, Japan; ^5^ Division of Diagnostic Pathology, Niigata Prefectural Central Hospital, Joetsu-Shi, Niigata 943-0147, Japan; ^6^ Division of Digestive and General Surgery, Graduate School of Medical and Dental Sciences, Niigata University, Chuo-Ku, Niigata 951-8510, Japan

**Keywords:** hepatocellular carcinoma, hepatic progenitor cell, epithelial cell adhesion molecule, lectin-reactive α-fetoprotein, des-γ-carboxy prothrombin

## Abstract

The high heterogeneity of hepatocellular carcinomas (HCCs) complicates stratification of HCC patients for treatment. Therefore, it is necessary to establish a comprehensive panel of HCC biomarkers related to tumour behaviour and cancer prognosis. Resected HCCs from 251 patients were stained for hepatic progenitor cell (HPC) markers epithelial cell adhesion molecule (EpCAM), neural cell adhesion molecule (NCAM), delta-like 1 homolog (DLK1), and cytokeratin 19 (CK19). Staining patterns were analysed for their prognostic association with relapse-free survival and overall survival. α-Fetoprotein (AFP), lectin-reactive α-fetoprotein (AFP-L3), and des-γ-carboxy prothrombin (DCP) were assessed as indicators of HPC protein expression. Expression pattern of HPC markers correlated with tumour malignancy indicated by high AFP/AFP-L3 serum levels, more frequent vascular invasion, and poorer tumour differentiation. EpCAM expression, DCP ≥300 mAU/ml, age ≥60, and Child-Pugh score grade B or C were independent prognostic factors of poor outcome and were used in a new scoring system for HCC prognosis after operation. Expression of two or more HPC markers was a significant predictor of poor HCC outcome and serum levels of AFP/AFP-L3 correlated with the expression of HPC proteins. Our study paved the way for further elucidation of the association among HPC markers, serum tumour markers, and HCC clinical outcome for precision medicine.

## INTRODUCTION

Liver cancers are the second most common cause of cancer-related death worldwide, and their incidence and mortality rates are on the rise [1, 2]. HCC accounts for 90% of primary liver cancers and represents a result of a highly complex and heterogeneous malignant process, usually within liver cirrhosis, triggered by genetic mutations [3]; risk factors include hepatitis B virus (HBV) and hepatitis C virus (HCV) infection, alcohol, and non-alcoholic steatohepatitis (NASH) [2]. The variety of genome mutational signatures and risk factors accounts for the high heterogeneity of HCCs not only among patients but also among tumours [4, 5], which is manifested by differences in morphology and malignant potential [6]. The diversity in HCC presentation complicates detailed classification of this cancer in terms of malignant behaviour, early recurrence, and drug resistance, negatively affecting HCC clinical management and resulting in poor prognosis [7]. Despite a variety of currently used therapeutic approaches, including surgical resection, liver transplantation, radiofrequency ablation, interventional therapy, chemotherapy, and molecular targeted therapy, some HCCs with high malignant potential are refractory to treatment. Therefore, it is necessary to establish an effective treatment algorithm for HCC cases with diverse molecular and genetic background.

The development of HCC molecular classification has paralleled the progress in genomic profiling technologies [8, 9]; however, although correlations between HCC subclasses and clinicopathological characteristics have been reported, more evidence is required to make informed clinical decisions for patients with HCCs [10]. For these reasons, in addition to genomic technologies, studies have focused on the expression of hepatic progenitor cell (HPC) markers, which may contribute to the heterogeneity of HCCs [11–13]. HPC-derived proteins have been used as predictive biomarkers of HCCs, and correlation between HPC protein levels and poor prognosis in HCC has been reported [14–16]. Many HPC markers such as epithelial cell adhesion molecule (EpCAM), neural cell adhesion molecule (NCAM), delta-like 1 homolog (DLK1), cytokeratin 19 (CK19), etc., are shared by some HCCs with malignant behaviour. The reason why HPC proteins are expressed in HCCs is still controversial. Some HCCs may arise from hepatic progenitor cells, while the others may start expressing HPC markers under the influence of the inflammatory environment around cancer cells [6, 14, 16–18]. It is thought that the expression of HPC markers in HCCs can provide a clue to predict patient outcome and serve as a potential target for precision medicine. However, there are many unsolved questions due to technical difficulties in evaluating HPC marker expression in HCC, which requires invasive tumour biopsy or surgery. For example, the contribution of individual HPC proteins expressed in HCC to the malignant process is currently unclear and the relationship between HPC markers and serum HCC markers such as α-fetoprotein (AFP), lectin-reactive AFP (AFP-L3), and des-γ-carboxy prothrombin (DCP) is unknown. AFP is the most representative HCC marker sometimes expressed by regenerating livers [19], AFP-L3 is an AFP isoform which can predict the malignant potential of HCC and, together with AFP, provide high sensitivity and specificity in HCC diagnosis [20–22], and DCP is an abnormal form of the clotting factor prothrombin which is another representative marker of HCC and often marks a distinct population of AFP-producing HCCs [23, 24]. Therefore, analysis of their correlation with HPC markers may help in more accurate prediction of HCC outcome.

In this study, we aimed to clarify the role of HPC markers in the heterogeneity of HCC manifestations by assessing the expression of four HPC markers, EpCAM, NCAM, DLK1, and CK19, in resected HCC tissues by immunohistochemistry and analysing their correlation with clinicopathological characteristics of HCC patients and their potential as prognostic factors for HCC patients after surgery. Furthermore, we determined the association of HPC marker expression profiles and HCC malignancy based on serum levels of tumour markers AFP and AFP-L3, and assessed the potential of AFP and AFP-L3 to predict the expression of HPC markers as an alternative non-invasive approach.

## RESULTS

### Clinicopathological characteristics of HCC patients and frequency of HPC marker expression

**Figure 1:**
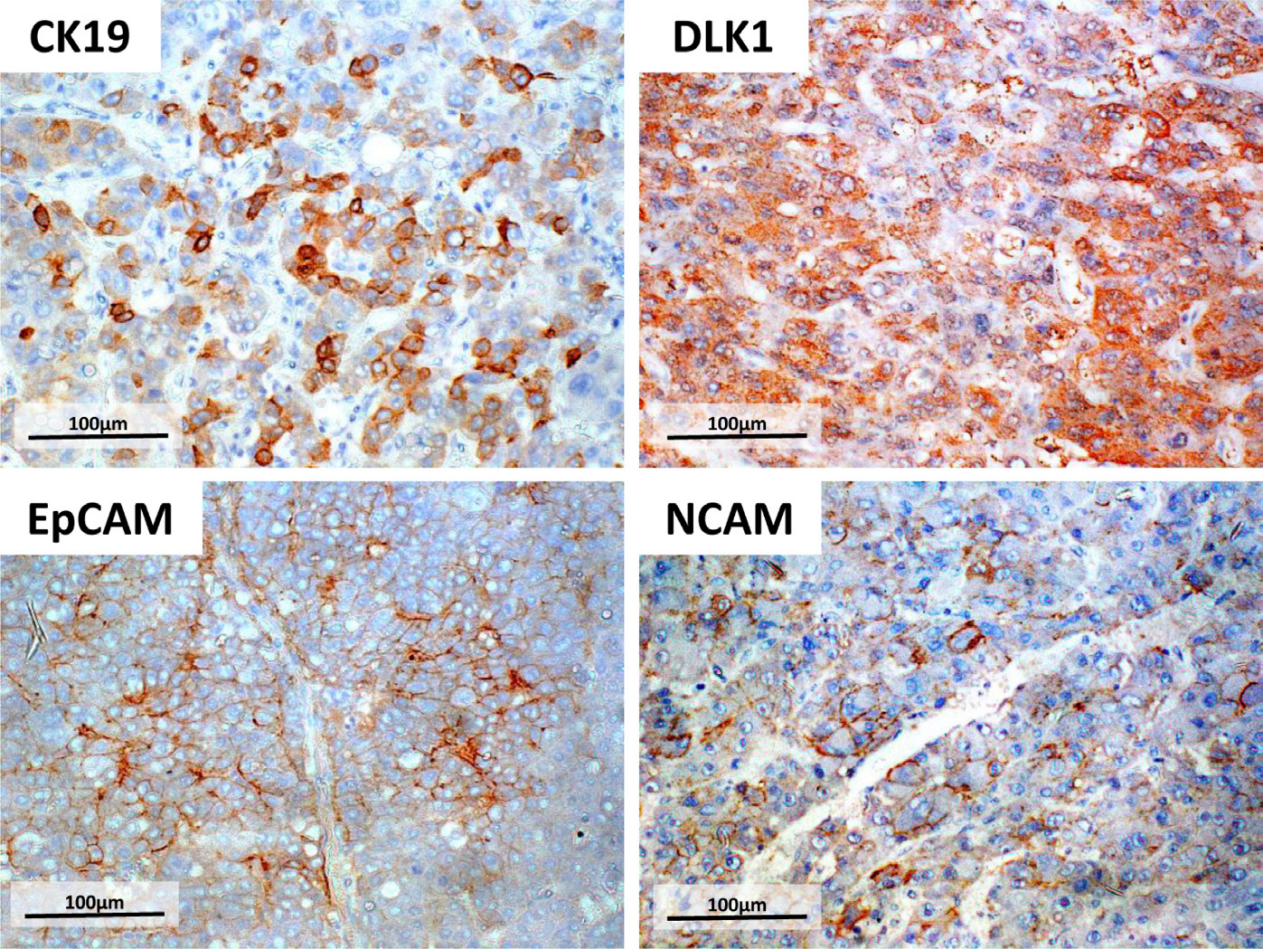
Immunohistochemistry of four HPC markers Surgically resected HCC specimens were fixed in formalin and stained for HPC markers DLK1, NCAM, EpCAM, or CK19 using specific antibodies. Tumours were considered positive for HPC marker expression if more than 5% of tumour cells in a single section were stained. Scale bar = 100 μm.

Patient clinicopathological characteristics are shown in Table 1. The majority of patients were 60 years or older (84.1%) and mostly men (78.9%). The predominant HCC etiology was liver disease caused by HCV (47.8%), followed by HBV (20.7%), alcohol (14.3%), and NASH (4.0%). Histologic tumour grades were mostly moderate (42.7%), followed by well/moderate (27.1%), well (14.7%), moderate/poor (13.5%), and poor (2.0%), and the majority of patients maintained liver functions as evidenced by Child-Pugh (C-P) scores (class A: 84.0%, class B: 15.2%, class C: 0.8%). Pathological analysis revealed vascular invasion, intrahepatic metastasis, and large tumours (≥5 cm) in 16.7%, 14.7%, and 19.5% patients, respectively. According to the TNM classification, most patients had tumours of the early stage (stage I, 60.0%), followed by those of stage II (22.7%), and stage III (18.3%). The percentage of patients with high (over cut-off) serum levels of AFP (cut-off value = 60 mg/dl), AFP-L3 (cut-off value = 20%), and DCP (cut-off value = 300 mAU/ml) was 21.5%, 26.0%, and 43.4%. HCC samples were considered positive for each HPC marker if more than 5% of tumour cells in a single section were stained (Figure 1); as a result, 18.3%, 7.1%, 14.3%, and 8.0% patients were found to have high levels of DLK1, NCAM, EpCAM, and CK19 in tumours, respectively.

**Table 1:** Clinicopathological features of patients with HCC

Variable	Overall cohort (*n* = 251)		Niigata university (*n* = 160)		Niigata prefectural central hospital (*n* = 91)	
	*n*	(%)	*n*	(%)	*n*	(%)
*n*	251	(100)	160	(63.7)	91	(36.3)
Age ≥60	211	(84.1)	126	(78.8)	85	(93.4)
Gender (males)	198	(78.9)	120	(75.9)	78	(85.7)
HPC marker						
DLK1 total	47	(18.7)	33	(20.6)	14	(15.4)
NCAM total	18	(7.1)	12	(7.5)	6	(6.6)
EpCAM total	36	(14.3)	22	(13.8)	14	(15.4)
CK19 total	20	(8.0)	13	(8.1)	7	(7.7)
Etiology						
HCV	120	(47.8)	78	(48.8)	42	(46.2)
HBV	52	(20.7)	42	(26.3)	10	(11.0)
Alcohol	36	(14.3)	27	(16.9)	9	(9.9)
NASH	10	(4.0)	8	(5.0)	2	(2.2)
Others	33	(13.2)	5	(3.1)	28	(30.8)
Histologic grade						
well	37	(14.7)	29	(18.1)	8	(8.8)
well/mod	68	(27.1)	51	(31.9)	17	(18.7)
mod	107	(42.7)	59	(36.9)	48	(52.7)
mod/poor	34	(13.5)	19	(11.9)	15	(16.5)
poor	5	(2.0)	2	(1.3)	3	(3.3)
Vascular invasion	42	(16.7)	28	(17.5)	14	(15.4)
Intrahepatic metastasis	37	(14.7)	30	(18.8)	7	(7.7)
Larger tumor size (≥5 cm)	49	(19.5)	24	(15.0)	25	(27.5)
TNM staging						
stage Ⅰ	148	(60.0)	100	(62.5)	48	(52.7)
stage Ⅱ	57	(22.7)	43	(26.9)	13	(14.4)
stage Ⅲ	46	(18.3)	17	(10.6)	29	(31.9)
Tumor marker						
AFP ≥60 mg/dl	54	(21.5)	39	(24.4)	15	(16.5)
AFP-L3 ≥20%	32	(26.0)	27	(25.0)	5	(33.3)
DCP ≥300 mAU/ml	102	(43.4)	61	(40.9)	41	(47.7)
Child-Pugh score						
5	150	(59.7)	109	(68.1)	41	(45.1)
6	61	(24.3)	35	(21.9)	26	(28.6)
7	20	(8.0)	9	(5.6)	11	(12.1)
8	16	(6.4)	5	(3.1)	11	(12.1)
9	2	(0.8)	1	(0.6)	1	(1.1)
10	2	(0.8)	2	(1.3)	0	(0.0)

### Correlation of HPC marker expression with the increase of serum AFP and AFP-L3 levels and the frequency of vascular invasion

Clinicopathological characteristics of patients positive for HPC markers are summarized in Table 2; patients negative for all HPC markers were used as control. First, we analysed the relationship between the expression of HPC markers and serum levels of tumour markers. Proportions of patients with AFP ≥60 mg/dl and AFP-L3 ≥20% were higher in HPC marker-positive groups (DLK1: *p* < 0.001 and *p* = 0.025; EpCAM: *p* < 0.001 and *p* = 0.011; and CK19: *p* < 0.001, respectively, compared to the control group). On the other hand, there was no difference between control and HPC-positive patients in the frequency of high serum DCP (≥300 mAU/ml) levels (Table 2). These results indicate that HPC marker expression is related to the increase of serum AFP and AFP-L3, but not DCP, in HCC patients.

**Table 2:** Clinicopathological characteristics of HCC patients positive for each HPC marker positive cases

Variable	All negative (*n* = 168)		DLK1 total (*n* = 47)		NCAM total (*n* = 18)		EpCAM total (*n* = 36)		CK19 total (*n* = 20)	
	*n*	(%)	*n*	(%)	*n*	(%)	*n*	(%)	*n*	(%)
*n*	168	(66.9)	47	(18.7)	18	(7.2)	36	(14.3)	20	(8.0)
Age≥60	141	(83.9)	38	(80.9)	14	(77.8)	32	(88.9)	18	(90.0)
Gender (males)	132	(78.6)	33	(70.2)	15	(83.3)	27	(75.0)	7	(35.0)
Etiology										
HCV	79	(47.0)	25	(53.2)	9	(50.0)	16	(44.5)	7	(35.0)
HBV	30	(17.9)	14	(29.8)	3	(16.7)	12	(33.3)	6	(30.0)
Alcohol	28	(16.7)	3	(6.4)	0	(0.0)	4	(11.1)	2	(10.0)
NASH	7	(4.2)	2	(4.3)	2	(11.1)	1	(27.8)	0	(0.0)
Others	24	(14.2)	3	(6.3)	4	(22.2)	3	(8.3)	5	(25.0)
Histologic grade										
well	32	(19.0)	2	(4.3)	1	(5.6)	3	(8.3)	0	(0.0)
well/mod	48	(28.6)	13	(27.8)	4	(22.2)	7	(19.4)	4	(20.0)
mod	62	(36.9)	23	(48.9)	11	(61.1)	19	(52.8)	11	(55.0)
mod/poor	24	(14.3)	6	(12.8)	2	(11.1)	5	(13.9)	3	(15.0)
poor	2	(1.2)	3	(6.4)	0	(0.0)	2	(5.6)	2	(10.0)
Vascular invasion	23	(13.7)	8	(17.0)	8	(44.4)	9	(25.0)	8	(40.0)
Intrahepatic metastasis	34	(20.2)	5	(10.6)	3	(16.7)	9	(25.0)	3	(15.0)
Larger tumor size (≥5 cm)	33	(19.6)	8	(17.0)	7	(38.9)	8	(22.2)	7	(35.0)
TNM staging										
stage Ⅰ	101	(60.1)	29	(61.7)	8	(44.4)	18	(50.0)	9	(45.0)
stageⅡ	35	(20.8)	12	(25.5)	5	(27.8)	11	(30.6)	5	(25.0)
stage Ⅲ	32	(19.1)	6	(12.8)	5	(27.8)	7	(19.4)	6	(30.0)
Tumor marker										
AFP ≥60 mg/dl	23	(13.7)	23	(48.9)	5	(27.8)	17	(47.2)	12	(60.0)
AFP-L3 ≥20%	13	(17.8)	13	(40.6)	5	(38.5)	9	(50.0)	9	(69.2)
DCP ≥300 mAU/ml	32	(20.3)	11	(25.0)	4	(23.5)	9	(29.0)	3	(18.8)
Child-Pugh score										
5	96	(57.2)	31	(66.0)	11	(61.1)	18	(50.0)	11	(55.0)
6	39	(23.2)	12	(25.5)	6	(33.3)	15	(41.6)	8	(40.0)
7	16	(9.5)	2	(4.3)	0	(0.0)	2	(5.6)	1	(5.0)
8	14	(8.3)	1	(2.1)	0	(0.0)	1	(2.8)	0	(0.0)
9	1	(0.6)	1	(2.1)	1	(5.6)	0	(0.0)	0	(0.0)
10	2	(1.2)	0	(0.0)	0	(0.0)	0	(0.0)	0	(0.0)

Then, we evaluated the association between the presence of HPC markers in HCCs and tumour differentiation or pathological vascular invasion, which indicate tumour malignancy. Well-differentiated HCCs were mostly HPC marker negative (*p* = 0.011), whereas HCCs with vascular invasion were mostly HPC marker-positive (NCAM: *p* = 0.003, CK19: *p* = 0.007) (Table 2), suggesting that tumour expression of HPC markers was related to the malignant potential of HCC.

### EpCAM expression, serum DCP, age, and Child-Pugh score are independent predictors of overall survival for HCC patients

**Figure 2:**
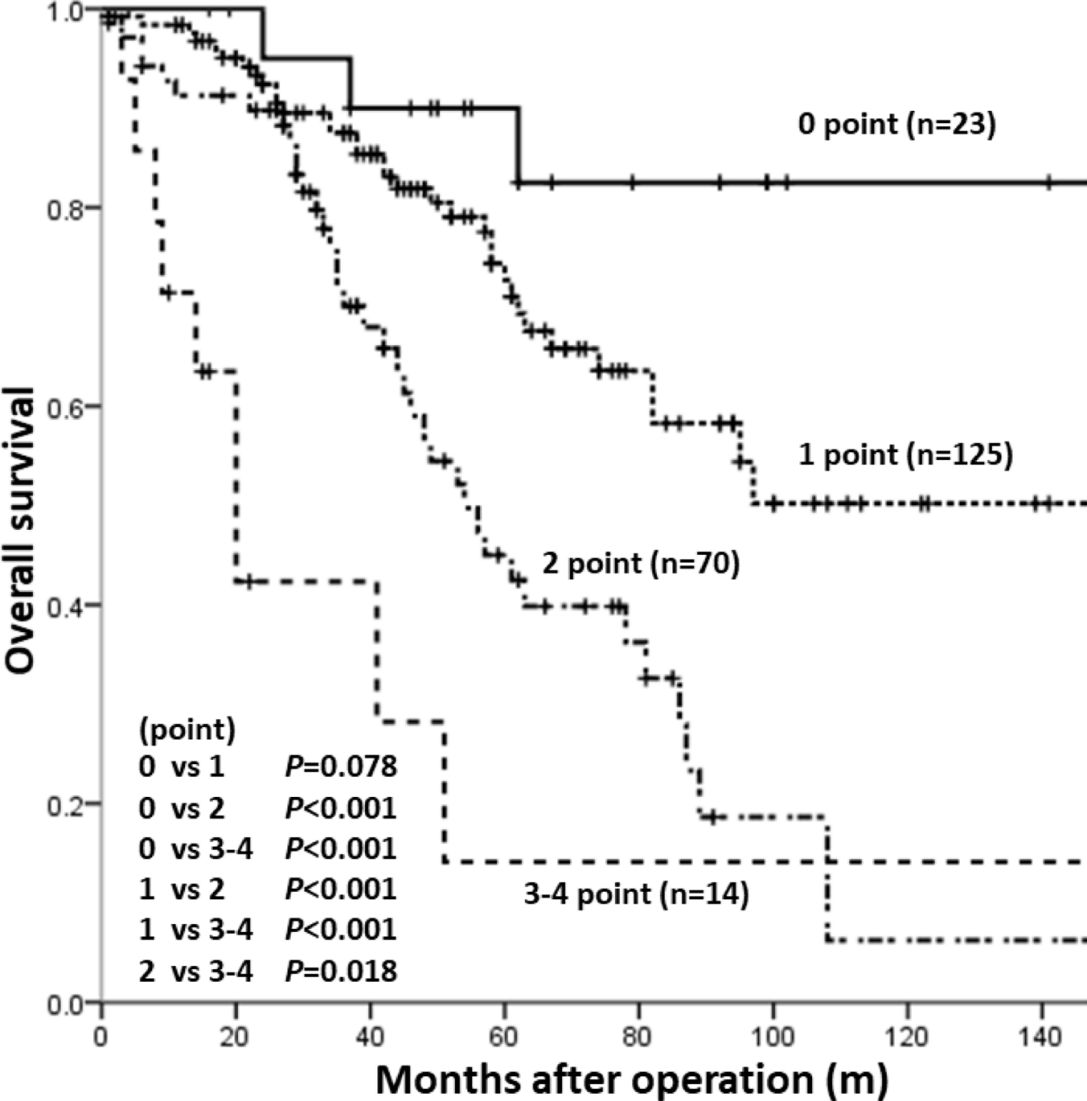
Kaplan-Meier analysis of OS in patient groups with different risk scores Patients were distributed into four groups based on the number of independent prognostic factors for OS (EpCAM expression, DCP ≥300 mAU/ml, age ≥60, and C-P score B or C, each given one score point). Patients with higher score had poorer prognosis compared to those with lower score.

As the expression of the four HPC markers in HCC was found to correlate with tumour malignancy, we examined the association of each marker with other clinicopathological factors linked to early recurrence or poor prognosis. For relapse-free survival (RFS), univariate analysis identified two HPC markers, DLK1 and EpCAM, and six clinicopathological characteristics (AFP ≥60 mg/dl, AFP-L3 ≥20%, DCP ≥300 mAU/ml, HCV infection, TNM stages II or III and intrahepatic metastasis) as prognostic factors. However, multivariate analysis confirmed only HCV infection (*p* = 0.025, HR = 1.485) as an independent predictor of early recurrence (Table 3). For overall survival (OS), two HPC markers, CK19 and EpCAM, and nine clinicopathological characteristics (AFP ≥60 mg/dl, AFP-L3 ≥20%, DCP ≥300 mAU/ml, age ≥60, C-P score B or C, vascular invasion, TNM stages II or III, and tumour size ≥5 cm and histological grade mod or mod/poor or poor) were revealed as prognostic factors by univariate analysis. However, only EpCAM expression (*p* = 0.006, HR = 2.237), DCP ≥300 mAU/ml (*p* = 0.033, HR = 1.839), age ≥60 (*p* = 0.011, HR = 2.731), and C-P score B or C (*p* < 0.001, HR = 2.246) were confirmed as independent predictors of poor outcome by multivariate analysis (Table 4). These four independent factors were employed to create a scoring model to predict OS. When the patients were stratified according to the number of factors they were positive for (0, 1, 2 and 3–4), Kaplan-Meir analysis of OS revealed that the higher score groups had poorer prognosis (Figure 2), indicating that EpCAM expression in HCCs together with DCP level, age, and C-P score could be used to reliably predict the outcome for HCC patients after surgery.

**Table 3:** Univariate and multivariate analyses of the association of clinicopathological features and HPC markers with relapse-free survival

		RFS (*n* = 220)					
		Univariate analysis			Multivariate analysis		
		*p* value	HR	95% CI	*p* value	HR	95% CI
HPC markers							
DLK1 total		**0.037**	1.522	1.025–2.258	0.358	1.234	0.788–1.930
NCAM total		0.201	1.452	0.820–2.572			
EpCAM total		**0.031**	1.651	1.048–2.602	0.091	1.509	0.936–2.433
CK19 total		0.746	1.125	0.550–2.301			
Tumor markers							
AFP (≥60 mg/dl)		**0.020**	1.816	1.239–2.663	0.263	1.305	0.819–2.082
AFP-L3 (≥20%)		**0.002**	2.184	1.335–3.572			
DCP (≥300 mAU/ml)		**0.002**	1.869	1.263–2.766	0.051	1.569	0.999–2.465
Clinical variables							
Age (≥60)		0.847	0.960	0.635–1.452			
Male gender		0.284	0.806	0.543–1.196			
Etiology	HCV	**0.034**	1.421	1.027–1.965	**0.025**	1.485	1.052–2.097
	HBV	0.873	0.968	0.647–1.447			
	Alcohol	0.763	0.927	0.565–1.519			
	NASH	0.411	1.376	0.643–2.943			
Histological grade		0.089	1.333	0.958–1.857			
(mod or mod-poor or poor)							
TNM staging (Ⅱor Ⅲ)		**0.033**	1.440	1.029–2.013	0.082	1.360	0.961–1.924
Vascular invasion		0.074	1.506	0.961–2.360			
Intrahepatic metastasis		**0.023**	1.651	1.072–2.543	0.077	1.489	0.957–2.317
Larger tumor size (≥5 cm)		0.780	0.936	0.589–1.489			
Child-Pugh score (B or C)		0.798	0.945	0.613–1.456			

**Table 4:** Univariate and multivariate analyses of the association of clinicopathological features and HPC markers with overall survival

		OS (*N* = 232) Univariate analysis			Multivariate analysis		
		*p* value	HR	95% CI	*p* value	HR	95% CI
HPC markers							
DLK1 total		0.606	1.150	0.675–1.959			
NCAM total		0.117	0.397	0.125–1.260			
EpCAM total		**0.001**	2.446	1.432–4.180	**0.006**	2.237	1.263–3.963
CK19 total		**0.017**	2.442	1.172–5.089	0.369	1.462	0.638–3.349
Tumor markers							
AFP (≥60 mg/dl)		**0.002**	2.070	1.311–3.269	0.222	1.383	0.822–2.326
AFP-L3 (≥20%)		**0.001**	3.130	1.572–6.233			
DCP (≥300 mAU/ml)		**<0.001**	2.384	1.487–3.821	**0.033**	1.839	1.049–3.223
Clinical variables							
Age (≥60)		**0.004**	3.001	1.418–6.351	**0.011**	2.731	1.258–5.928
Male gender		0.283	0.747	0.439–1.272			
Etiology	HCV	0.157	1.366	0.886–2.107			
	HBV	0.050	0.531	0.282–1.001			
	Alcohol	0.501	1.244	0.659–2.350			
	NASH	0.895	0.925	0.292–2.934			
Histological grade		**0.011**	1.756	1.136–2.715	0.472	1.190	0.740–1.913
(mod or mod-poor or poor)							
TNM staging (Ⅱor Ⅲ)		**0.001**	2.338	1.513–3.613	0.072	1.708	0.952–3.062
Vascular invasion		**<0.001**	2.791	1.641–4.746	0.161	1.579	0.833–2.994
Intrahepatic metastasis		0.076	1.604	0.952–2.702			
Larger tumor size (≥5 cm)		**0.029**	1.828	1.065–3.138	0.796	1.087	0.578–2.043
Child-Pugh score (B or C)		**0.003**	2.046	1.268–3.302	**<0.001**	2.246	1.349–3.741

### Simultaneous expression of different HPC markers correlates with HCC malignant potential

**Figure 3:**
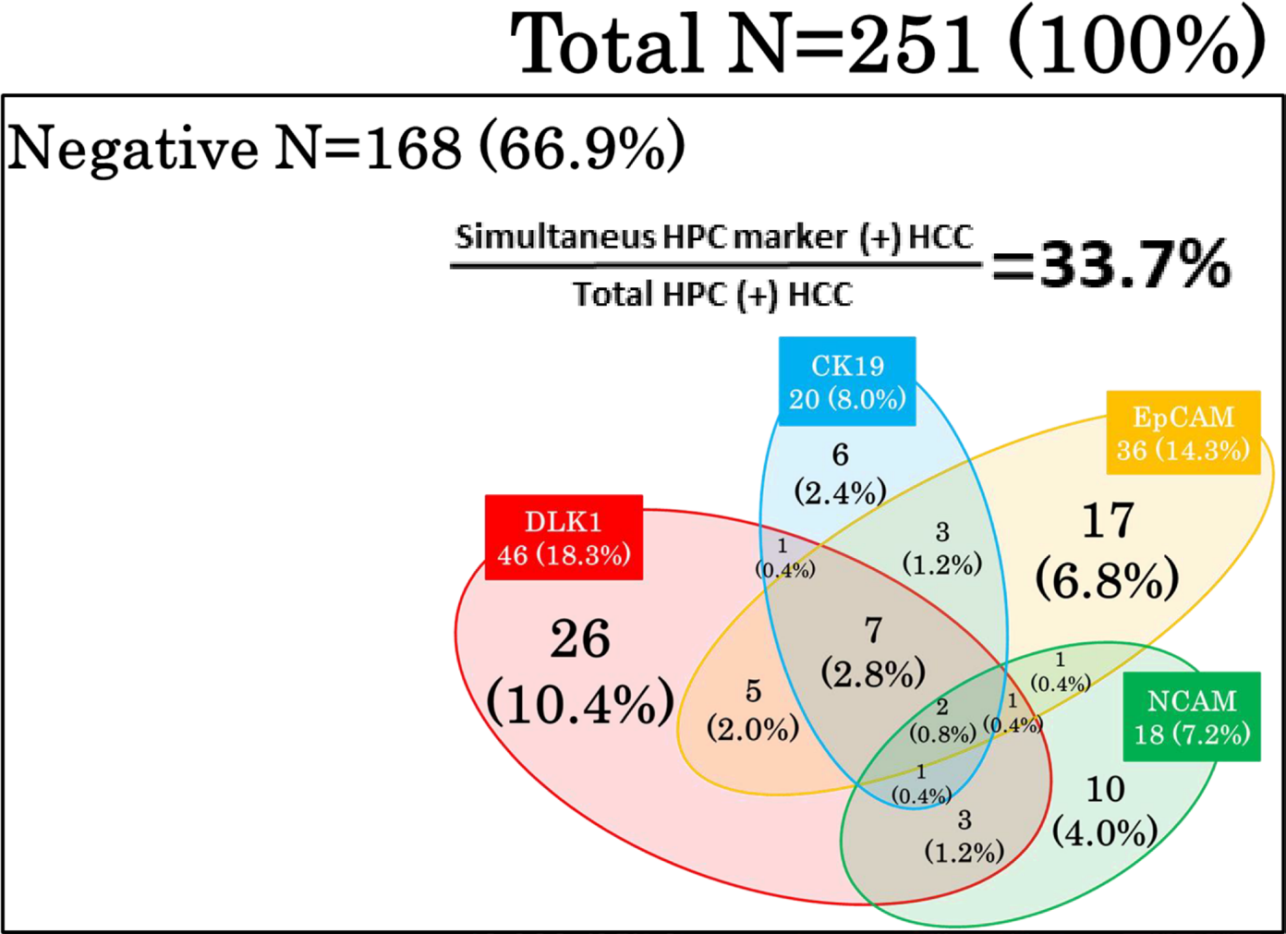
Expression pattern of HPC markers in HCCs A Venn diagram illustrates high heterogeneity of HCCs which expressed different combinations of HPC markers.

The relationship between HPC markers in HCC was analysed using a Venn diagram (Figure 3), which illustrated high heterogeneity of HCCs in the expression of HPC markers. For example in the EpCAM-positive group which was shown to have poor prognosis, many tumours also expressed other HPC markers in different combinations, indicating that EpCAM-positive HCCs are highly heterogeneous regarding the presence of the other HPC markers. The frequencies of a single marker expression were 10.4% (DLK1), 6.8% (EpCAM), 4.0% (NCAM), and 2.4% (CK19), indicating that other HPC marker-positive HCCs (approximately 33.7% of the total number of HPC marker-positive tumours) expressed two or more HPC proteins. These data suggest the necessity of evaluating different combinations of HPC biomarkers in HCC.

To analyse the impact of different HPC protein combinations in HCC, we divided the patients into groups expressing one, two or more (≥2 M), and three or more (≥3 M) HPC markers (Table 5). Compared to the patient stratification which did not consider the number of HCC-expressed HPC markers (Table 2), there were significant differences in the association of HPC markers with clinicopathological characteristics (Table 5). Thus, the frequency of poorly differentiated HCC was the highest in the ≥3 M group (18.2%), followed by the ≥2 M group (8.3%), and single-marker groups (3.8% for DLK1, and 0% for NCAM, EpCAM, and CK19). Similarly, the proportions of patients with AFP ≥60 mg/dl and AFP-L3 ≥20% were the highest in the ≥3 M group (81.8% and 75.0%, respectively), followed by the ≥2 M group (62.5% and 62.5%, respectively), whereas in single-marker groups these numbers were much lower (Table 5). On the other hand, there was no significant difference between single-marker and ≥2 M and ≥3 M groups in the proportion of patients with DCP ≥300 mAU/ml. These data indicate that the number of HPC proteins expressed in HCC is associated with increased serum levels of AFP and AFP-L3 and poorer differentiation.

**Table 5:** Clinicopathological characteristics of patients with HCCs positive for single and multiple HPC markers

Variable	All negative (*n* = 168)		DLK1 single (*n* = 26)		NCAM single (*n* = 10)		EpCAM single (*n* = 17)		CK19 single (*n* = 6)		≥2 marker (*n* = 24)		≥3 marker (*n* = 11)	
	*n*	(%)	*n*	(%)	*n*	(%)	*n*	(%)	*n*	(%)	*n*	(%)	*n*	(%)
*n*	168	(66.9)	26	(10.4)	10	(4.0)	17	(6.8)	6	(2.4)	24	(9.6)	11	(4.4)
Age ≥60	141	(83.9)	21	(80.8)	8	(80.0)	15	(88.2)	6	(100)	21	(87.5)	9	(81.8)
Gender (males)	132	(78.6)	20	(76.9)	8	(81.0)	16	(94.1)	6	(100)	15	(62.5)	5	(45.5)
Etiology														
HCV	79	(47.0)	15	(57.7)	6	(60.0)	8	(47.1)	2	(33.3)	11	(45.8)	4	(36.4)
HBV	30	(17.8)	8	(30.8)	2	(20.0)	5	(29.4)	0	(0.0)	7	(29.2)	5	(45.4)
Alcohol	28	(16.7)	2	(7.7)	0	(0.0)	4	(23.5)	1	(16.7)	1	(4.2)	0	(0.0)
NASH	7	(4.2)	0	(0.0)	1	(10.0)	0	(0.0)	0	(0.0)	2	(8.3)	0	(0.0)
Others	24	(14.3)	1	(3.8)	1	(1.00)	0	(0.0)	3	(50.0)	3	(12.5)	2	(18.2)
Histologic grade														
well	32	(19.0)	1	(3.8)	1	(10.0)	2	(11.8)	0	(0.0)	1	(4.2)	0	(0.0)
well/mod	48	(28.6)	8	(30.9)	2	(20.0)	3	(17.6)	1	(16.7)	6	(25.0)	2	(18.2)
mod	62	(36.9)	13	(50.0)	6	(60.0)	10	(58.8)	4	(66.7)	12	(50.0)	5	(45.4)
mod/poor	24	(14.3)	3	(11.5)	1	(10.0)	2	(11.8)	1	(16.7)	3	(12.5)	2	(18.2)
poor	2	(1.2)	1	(3.8)	0	(0.0)	0	(0.0)	0	(0.0)	2	(8.3)	2	(18.2)
Vascular invasion	23	(13.7)	2	(7.7)	3	(30.0)	4	(23.5)	3	(50.0)	7	(29.2)	3	(27.3)
Intrahepatic metastasis	34	(20.2)	2	(7.7)	1	(10.0)	6	(35.3)	1	(16.7)	3	(12.5)	2	(18.2)
Larger tumor size (≥5 cm)	33	(19.6)	2	(7.7)	2	(20.0)	2	(11.8)	2	(33.3)	8	(33.3)	3	(27.3)
TNM staging														
stageⅠ	101	(60.1)	19	(73.1)	5	(50.0)	9	(52.9)	2	(33.3)	12	(50.0)	5	(45.5)
stageⅡ	35	(20.8)	5	(19.2)	3	(30.0)	6	(35.3)	2	(33.3)	6	(25.0)	4	(36.4)
stage Ⅲ	32	(19.1)	3	(11.5)	2	(20.0)	2	(11.8)	2	(33.3)	6	(25.0)	2	(18.1)
Tumor marker														
AFP ≥60 mg/dl	23	(13.7)	8	(30.8)	0	(0.0)	6	(35.3)	2	(33.3)	15	(62.5)	9	(81.8)
AFP-L3 ≥20%	13	(17.8)	4	(22.2)	1	(16.7)	3	(42.9)	1	(33.3)	10	(62.5)	6	(75)
DCP ≥300 mAU/ml	32	(20.3)	6	(23.1)	1	(10.0)	5	(31.3)	1	(20.0)	5	(25.0)	3	(33.3)
Child-Pugh score														
5	96	(57.2)	20	(76.9)	8	(80)	10	(58.8)	5	(83.3)	11	(45.8)	5	(45.5)
6	39	(23.2)	4	(15.5)	2	(20)	5	(29.4)	0	(0.0)	11	(45.8)	6	(54.5)
7	16	(9.5)	1	(3.8)	0	(0.0)	1	(5.9)	1	(16.7)	1	(4.2)	0	(0.0)
8	14	(8.3)	1	(3.8)	0	(0.0)	1	(5.9)	0	(0.0)	0	(0.0)	0	(0.0)
9	1	(0.6)	0	(0.0)	0	(0.0)	0	(0.0)	0	(0.0)	1	(4.2)	0	(0.0)
10	2	(1.2)	0	(0.0)	0	(0.0)	0	(0.0)	0	(0.0)	0	(0.0)	0	(0.0)

**Figure 4:**
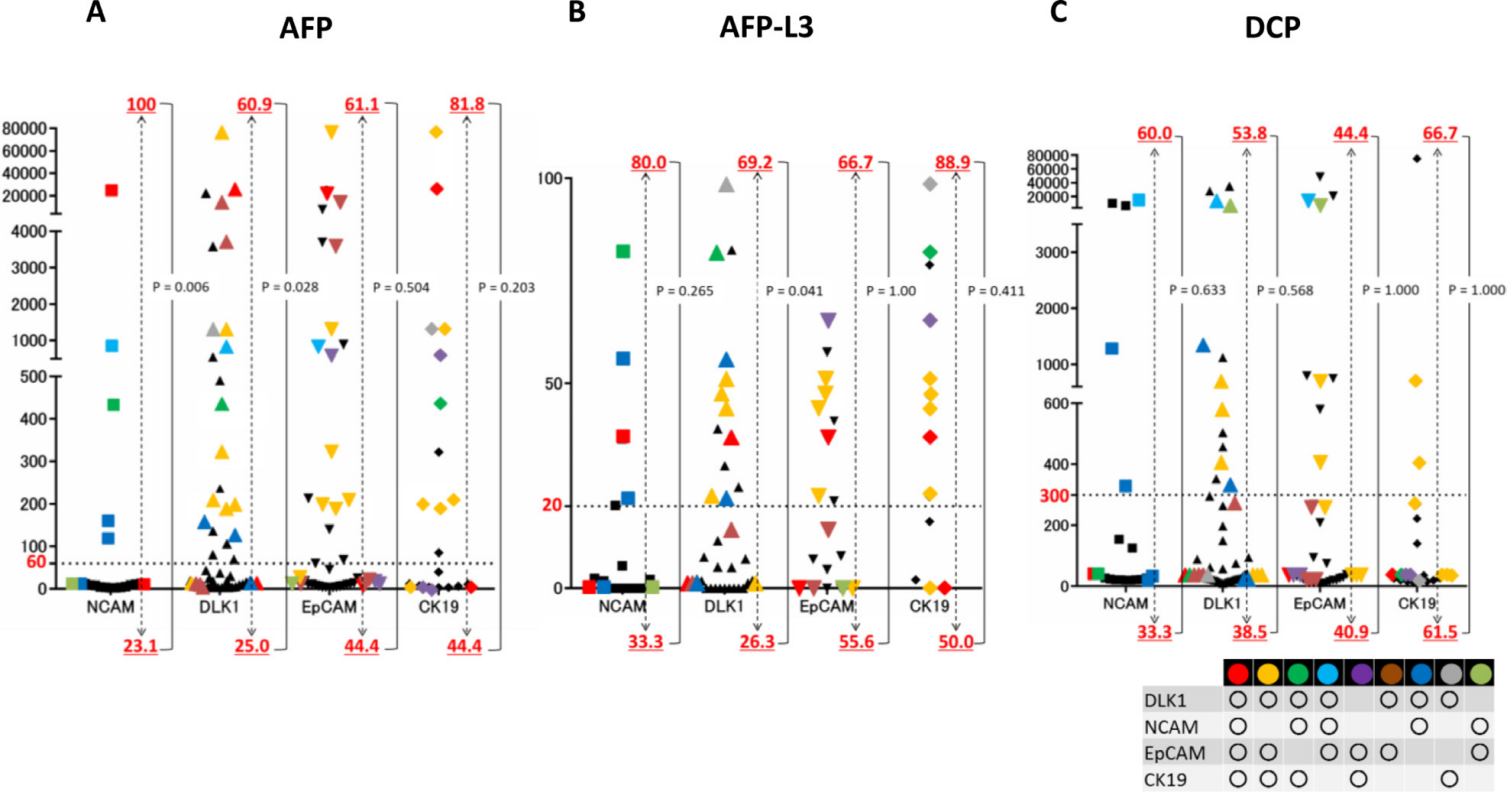
Correlation of serum tumour markers with the expression of HPC proteins in HCC Levels of serum tumour markers in patients with HCCs expressing one HPC protein (black symbols) or two or more proteins in different combinations (coloured symbols). The frequency of HCCs positive for two or more HPC markers is indicated by red numbers above and below the dotted line (cut-off values); the top row shows the percentage of patients with high serum levels of AFP (≥60 mg/dl), AFP-L 3 (≥20%), and DCP (≥300 mAU/ml) and the bottom row – those with low levels (<60 mg/dl, <20%, and <300 mAU/ml, respectively). Most patients with high serum levels of AFP/AFP-L3 expressed two or more HPC markers.

To further clarify the relationship between serum tumour markers and the expression pattern of HPC markers, we next determined the proportion of patients expressing ≥2 HPC markers in the group with increased levels of serum tumour markers (Figure 4). Among the total population of patients positive for HPC markers (regardless of their number), patients with AFP ≥60 mg/dl tended to express ≥2 markers, especially NCAM (*p* = 0.006) and DLK1 (*p* = 0.028) (Figure 4A); the same tendency was shown in patients with AFP-L3 ≥20%, most of which expressed DLK1 (*p* = 0.041) (Figure 4B) (percentages of patients with elevated AFP and AFP-L3 levels expressing a particular HPC marker together with any other marker (s) are shown as red numbers above the dotted lines). However, no difference in the HPC marker expression pattern was detected between patients with low and high serum DCP (Figure 4C). These data indicate that the elevation of serum AFP/AFP-L3 levels correlate with the expression of two or more HPC markers in HCC, suggesting that these tumours had a more severe malignant phenotype compared to those positive for a single HPC marker.

### Simultaneous expression of HPC markers is a significant risk factor for early recurrence and poor prognosis

**Figure 5:**
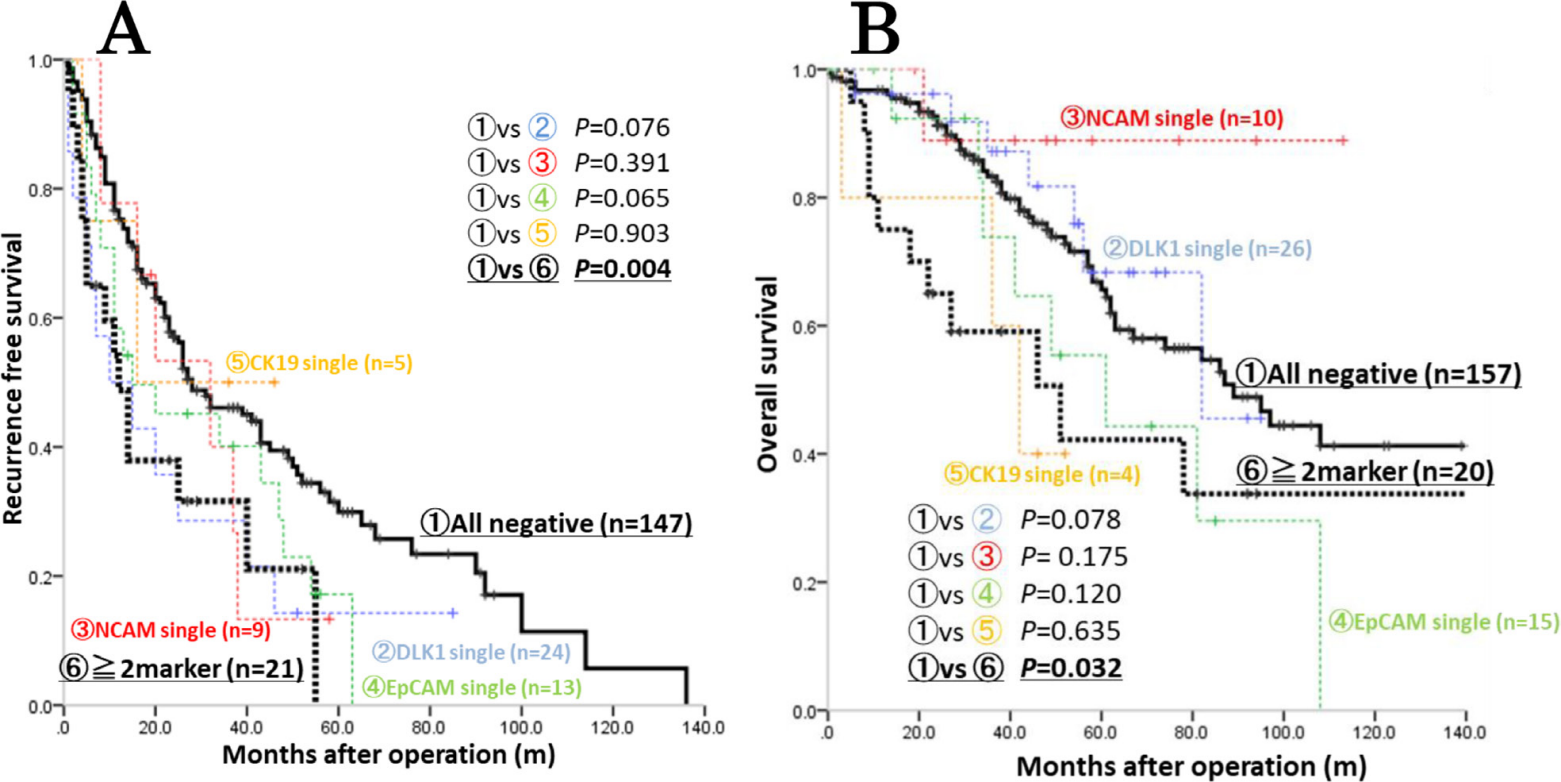
Kaplan-Meier analysis of survival among patients with HCCs positive and negative for HPC markers Patients with HCCs expressing two or more HPC markers (≥2 markers) had significantly shorter RFS (**A**) and OS (**B**) compared to control (all negative) group (*p* = 0.004 and *p* = 0.032, respectively). Each colour distinguishes each single HPC marker expression (red: NCAM, blue: DLK1, green: EpCAM, yellow: CK19).

To confirm the notion about increased malignancy of HCCs expressing more than one HPC marker, we analysed the association of the HPC protein expression pattern with HCC prognosis and early recurrence using Kaplan-Meier analysis. While there was no significant difference in RFS or OS between HPC marker-negative and single HPC marker-positive groups, they were significantly shorter for patients with tumours expressing two or more markers (RFS: *p* = 0.004; OS: *p* = 0.032) (Figure 5). These data indicate that simultaneous expression of HPC marker was a significant risk factor of early recurrence and poor prognosis in HCC patients.

### Combination of serum AFP and AFP-L3 levels can predict simultaneous expression of HPC markers in HCC

**Figure 6:**
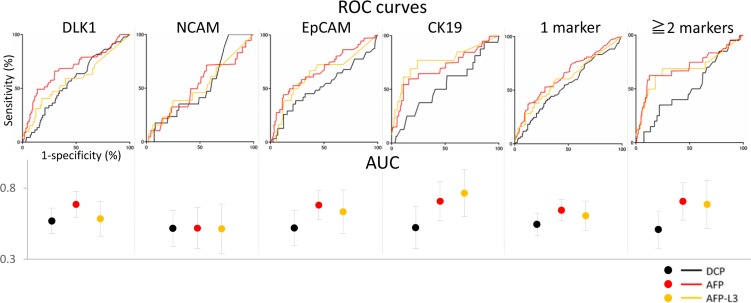
Prediction of HPC marker expression in HCCs based on serum levels of tumour markers ROC curves for sensitivity of serum tumour markers DCP (black), AFP (red), and AFP-L3 (yellow). AUC (specificity) is shown with 95% CI (black: DCP; red: AFP; yellow: AFP-L3). AFP and AFP-L3 were identified as reliable predictors of HCC positivity for HPC markers (except NCAM).

Given that the expression of HPC markers, especially two or more, was related to increased AFP/AFP-L3 levels, we examined whether the expression of a particular HPC marker (DLK1, NCAM, EpCAM, and CK19), one or more HPC markers (≥1 marker), and two or more markers (≥2 markers) could be predicted based on serum levels of AFP, AFP-L3, and DCP (Figure 6, Table 6). In case of AFP, ROC analysis revealed that the AUC for CK19, DLK1, EpCAM, and ≥1 marker and ≥2 marker groups was 0.708, 0.686, 0.681, 0.645, and 0.707, respectively, indicating high predictive power of serum AFP regarding the expression pattern of HPC markers. Similar results were obtained for serum AFP-L3, when the AUC for CK19, EpCAM, and ≥2 marker groups was 0.765, 0.634, and 0.686, respectively. However, neither AFP nor AFP-L3 could predict NCAM expression, whereas DCP showed no predictive power for HPC markers. Cumulatively, these data demonstrate that serum AFP and AFP-L3 levels could be used to predict the expression of HPC markers, especially of CK19 and a combination of two or more markers, in HCC.

**Table 6:** AUC, sensitivity, and specificity of serum tumor markers for prediction of HPC marker expression

	DCP			AFP			AFP-L3		
	(cut point 300 mAU/ml)			(cut point 60 mg/ml)			(cut point 20%)		
	AUC	sensitivity	specificity	AUC	sensitivity	specificity	AUC	sensitivity	specificity
DLK1	0.569	25	79.1	0.686	48.9	84.8	0.585	40.6	79.1
NCAM	0.518	23.5	78.4	0.518	72.2	21	0.514	38.5	75.5
EpCAM	0.519	29	79.4	0.681	47.2	82.8	0.634	50	78.1
CK19	0.522	81.3	21.9	0.708	60	81.8	0.765	69.2	79.1
HPC marker	0.545	24.7	79.8	0.645	37.4	86.3	0.606	38	82.2
≥2 marker	0.509	25	78.6	0.707	62.5	82.8	0.686	62.5	79.4

Next, we determined the sensitivity and specificity of AFP (≥60 mg/dl) and AFP-L3 (≥20%) in predicting the expression of HPC markers. The sensitivity of both AFP and AFP-L3 was about 50%; however, their specificity was high (except for NCAM): over 80% for AFP and close to 80% for AFP-L3 (Figure 6, Table 6). These date suggest that AFP and AFP-L3 can serve as indicators of HPC expression in HCCs.

**Figure 7:**
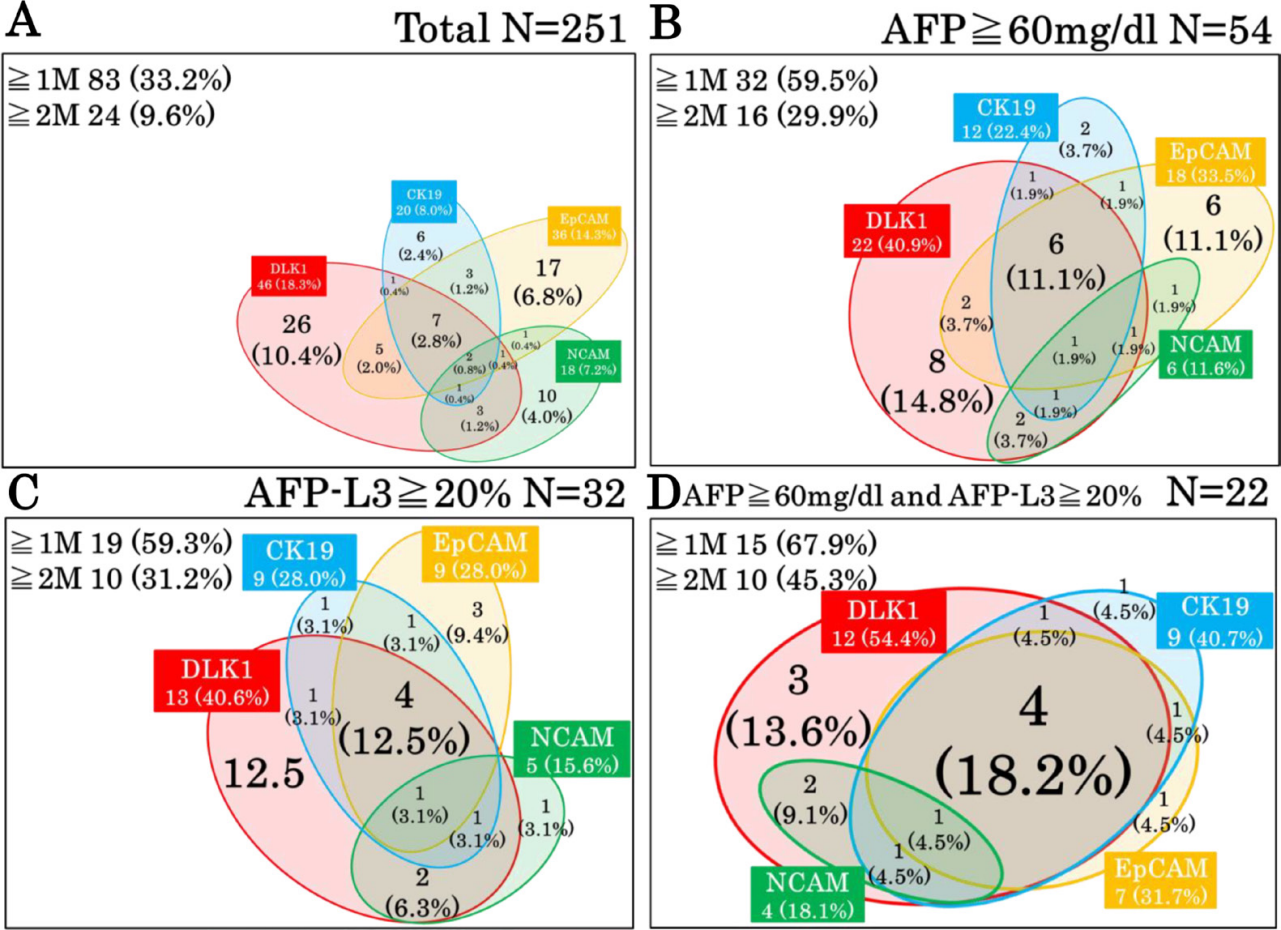
Relationships among HPC markers in HCCs depending on the levels of serum tumour markers (**A**) All analysed HCC patients. (**B**) HCC patients with high serum levels of AFP (≥60 mg/dl). (**C**) HCC patients with high serum levels of AFP-L3 (≥20%). (**D**) HCC patients with high serum levels of both AFP and AFP-L3. ≥1 M and ≥2 M indicate HCCs positive for one or more and two or more HPC markers, respectively.

Finally, we examined the possibility to predict the expression pattern of HPC markers in HCC as a factor related to tumour malignancy based on serum AFP and AFP-L3 levels using a Venn diagram. Among the analysed HCCs, the frequency of tumours expressing one or more HPC markers (≥1 M) and two or more HPC markers (≥2 M) were 33.2% and 9.6%, respectively (Figure 7A). However, when we considered HCC patients with AFP ≥60 mg/dl or AFP-L3 ≥20%, the expression frequency of one or more HPC markers (≥1 M) and two or more HPC markers (≥2 M) in HCC increased to 59.5% and 29.9% for AFP (Figure 7B) and 59.3% and 31.2% for AFP-L3, respectively (Figure 7C), and further increased to 67.9% and 45.3%, respectively, for patients with high serum levels of both AFP and AFP-L3 (Figure 7D). These data clearly indicate that the expression pattern of HPC markers in HCC could be more accurately predicted based on serum levels of both AFP and AFP-L3.

## DISCUSSION

In this study, we analysed the association between clinicopathological characteristics of HCC patients and tumour expression of four HPC markers, EpCAM, NCAM, CK19, and DLK1. Our results indicate that EpCAM expression together with serum DCP, age, and C-P scores can serve as an independent prognostic factor for patients after HCC resection and that the level of serum tumour markers AFP and AFP-L3 can predict the expression of HPC markers in HCC, thus serving as a non-invasive diagnostic method.

The heterogeneity of HCCs has been extensively investigated, particularly focusing on genetic background as an HCC risk factor [25] Potential therapeutic targets in HCC, such as β-catenin, tumour protein p53, and axin 1 were identified by Schulze *et al.* [10]. They also reported that mutations in the telomerase reverse transcriptase (*TERT*) promoter were related to early stages, whereas the amplification of genes encoding fibroblast growth factors FGF3, FGF4, and FGF19 or cyclin-D1 and polymorphisms in p53 and cyclin-dependent kinase inhibitor 2A genes were implicated in advanced stages of aggressive tumours. Genetic analysis also provided a map of virus integration sites in HCC, and it has been reported that mutations in *TERT*, myeloid/lymphoid leukaemia 4 (*MLL4*), G1/S-specific cyclin-E1 and 1110 (*CCNE1* and *CCNE1110*), histone-lysine N-methyltransferase (*KMTB2*), and cyclin-A2 (*CCNA2*) genes were related to HBV- and adeno-associated virus type 2-induced carcinogenesis [26, 27]. In addition, the tumour microenvironment also contributes to HCC heterogeneity. Thus, it was shown that cancer cells growing in cirrhotic conditions, interacting with stromal cells, and/or engaging extracellular matrix components have different potential for tumorigenesis and cancer spread [28, 29] and that immune cells such as macrophages and T cells can affect malignant behaviour of cancer cells. These factors can present potential targets for precision medicine [4, 30].

There is also evidence that liver tumours express HPC markers which contribute to HCC heterogeneity and serve as indicators of HCC malignant phenotype in clinical practice. Thus, precision medicine should be based on the integration of clinicopathological, genetic, and biochemical characteristics of HCC patients, including the expression of HPC proteins. Therefore, in this study we assessed HCC heterogeneity according to the presence of HPC markers DLK1, NCAM, EpCAM, and CK19 in the tumour, and analysed their association with HCC prognosis.

Univariate analysis indicated that the expression of DLK1 and EpCAM can serve as a prognostic factor of RFS, whereas CK19 and EpCAM can predict OS, and EpCAM expression was confirmed as an independent prognostic factor of OS by multivariate analysis. These data are consistent with previous reports that patients with EpCAM-positive HCCs had enhanced tumour growth, higher frequency of portal vein invasion, and significantly shorter survival [31–34]. Although some studies suggest the relationship between DLK1 or CK19 and tumour malignant behaviour, they were not confirmed as independent prognostic factors of OS in our study [35–37]. The scoring model based on the combination of four independent prognostic factors (DCP ≥300 mAU/ml, age ≥60, C-P score grade B or C, and EpCAM expression) accurately predicted poor outcome after surgery, indicating prognostic utility of HPC markers. Similar to our model, HPC-related markers have been proved reliable prognostic factors for HCC in other studies. Thus, Yang *et al.* [15] have reported that their scoring system including CD133, CD44, Nestin, and microvessel density was more accurate in HCC prognosis than that based on other clinicopathological parameters, including single HPC marker expression. Our scoring model is simple as it is based on three easily measurable clinical factors and one HPC marker, and, therefore, may be useful in clinical practice. While we assessed HPC marker expression in resected HCC specimens by immunohistochemistry, a new method for testing HPC markers in circulating tumour cells obtained by liquid biopsy is being developed, and the relationship between EpCAM expression in liquid biopsy samples and poor prognosis in HCC has been reported [38, 39]. Because we examined a limited number of HPC markers, only EpCAM expression was defined as an independent prognostic factor; however, other HPC markers may also be identified as such factors in HCC and may be used to build a more detailed scoring system to predict survival of HCC patients. At the moment, our results strongly indicate that EpCAM expression in HCCs is an indicator of increased tumour malignancy and poor prognosis.

Previous clinical studies have also reported an association between the expression of particular HPC markers and tumour malignant behaviour [31, 37, 40, 41] which is consistent with our data on EpCAM. However, HPC markers are often co-expressed in various combinations (Figure 3), suggesting that simultaneous expression of different HPC markers should be analysed in order to understand the heterogeneity among HCCs. Indeed, here we observed that EpCAM was often expressed in HCC together with the other HPC markers; therefore, to get more insight into the relationship between HCC malignancy and HPC protein expression pattern, we classified our patients based on the presence of none, one, two or more, and three or more HPC markers (Table 5). Our results indicated that the expression of two or more HPC markers in HCC correlated with poorer tumour differentiation and increase in serum AFP/AFP-L3, indicating enhanced malignancy which was confirmed by Kaplan-Meier analysis showing shorter OS and RFS for patients with HCC positive for two or more HPC markers. Although some previous studies showed the association of poor HCC prognosis with the expression of single HPC markers [42–44] there are few reports on such an association for a combination of HPC markers [15]. Our results clearly demonstrated high malignant potential of HCCs expressing two or more HPC markers, emphasizing the importance of evaluating a comprehensive panel of HPC markers to reliably predict HCC outcome. Because of a limited number of patients, we could not perform a more detailed classification according to different combinations of HPC markers; therefore, studies based on larger patient cohorts are required to further elucidate the role of the HPC protein expression pattern in HCC malignancy and prognosis.

However, although we established the prognostic utility of multiple HPC markers in HCC, their expression can be assessed only in tumour biopsy or surgical specimens obtained by invasive procedures. Therefore, to develop an alternative method to detect HPC markers in HCCs, we analysed their association with conventional serum tumour markers. ROC analysis revealed that serum AFP and AFP-L3 levels showed high specificity in predicting the expression of HPC markers in HCC, especially two or more of them, and can be used to segregate HPC-negative tumours. A previous study used genomic profiling to divide HCCs into non-proliferating and proliferating subclasses [45]; the latter comprises aggressive cancers with progenitor cell-like characteristics, moderate to poor cell differentiation by histology, frequent vascular invasion, higher AFP levels, and poorer prognosis [11, 42]. Given close association between simultaneous presence of HPC markers in HCCs and AFP/AFP-L3 levels revealed in our study, it is conceivable that HCCs expressing multiple HPC markers belong to the proliferating subclass. In addition, we identified serum DCP as an independent predictor of poor outcome unrelated to HPC markers, suggesting its contribution to HCC heterogeneity and prognostic value for HCC outcome.

A limitation of this study is that we analysed limited number of HPC markers; thus, combination with other markers will elucidate the complex heterogeneity and detailed classification of HCC. Furthermore, we used immunohistochemistry; however, combination with real-time PCR analysis may result in a more objective analysis, and further analysis will provide more robust data. We consider that combination with HPC marker, tumor marker, mutated gene, and clinical treatment data will facilitate development of future precision medicine.

## MATERIALS AND METHODS

### Patients and specimens

**Figure 8:**
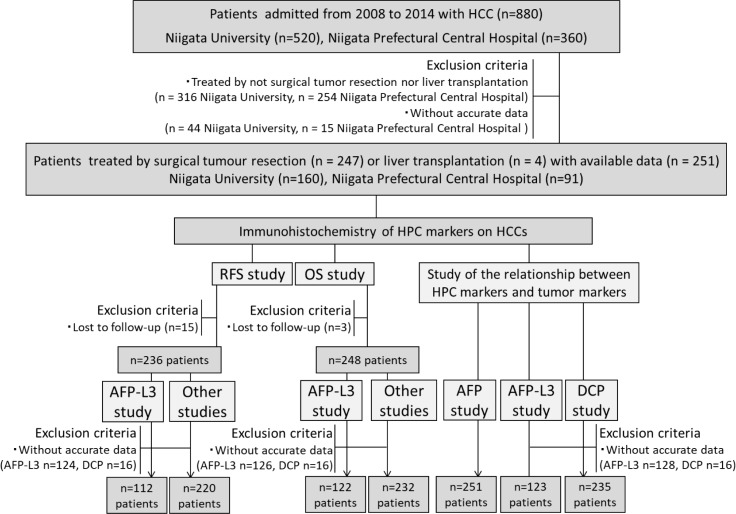
Flow chart of the study The initial cohort included 880 HCC patients admitted from 2008 to 2014 to Niigata University (*n =* 520) and Niigata Prefectural Central Hospital (*n =* 360). The data were available for a total of 251 patients, who were treated by surgical tumour resection (*n =* 247) or liver transplantation (*n =* 4). Patients lost to follow-up and/or without accurate data on serum AFP, AFP-L3, or DCP levels were excluded as necessary in each stage of the study.

A total of 251 patients with HCC, which were treated by surgical tumour resection (*n =* 247), including minimal surgery such as enucleation (*n =* 36, C-P score B) or liver transplantation (*n =* 4, C-P score C) from 2008 to 2014 in Niigata University Hospital (*n =* 160) and Niigata Prefectural Central Hospital (*n =* 91) were retrospectively analysed. This study was approved by the Institutional Review Board (IRB) of both hospitals. HCC was diagnosed by CT or MRI within two months and serum analysis was performed within one week before operation. Patients lost to follow-up and/or without accurate clinical data were excluded as necessary in each experimental step, as shown in the diagram of the study protocol (Figure 8).

### Immunohistochemistry

HCC specimens were analysed for the expression of four HPC markers using mouse monoclonal antibodies against EpCAM and NCAM (Santa Cruz biotechnology, Dallas, TX, USA), CK19 (Dako, Santa Clara, CA, USA), and DLK1 (kindly provided by CHIOME Bioscience Inc.). We selected one slide with a representative histological type in each case. HCC tissues were fixed in 10% formalin, embedded in paraffin, cut into 5-μm sections, and mounted on glass slides. Sections were deparaffinized and treated for antigen retrieval in 10 mM sodium citrate buffer, pH 6.0, in microwave for 8 min (EpCAM) and 20 min (NCAM and CK19); DLK1 was retrieved by the treatment in 10 mM Tris-HCl/1 mM EDTA, pH 9.0, for 17 min. Endogenous peroxidase activity was blocked with 3 % hydrogen peroxide in methanol (Wako, Japan) for 10 min at room temperature, and sections were incubated overnight with primary antibodies (EpCAM and NCAM: 1/100; CK19: 1/50; DLK1: 1/700) in Antibody Diluent Reagent Solution (Thermo Fisher Scientific, Waltham, MA, USA). Slides were then stained using the Vectastain ABC kit (Vector Laboratories, Burlingame, CA, USA) and DAB TRIS tablets (Muto Pure Chemicals, Japan) for EpCAM, NCAM, and CK19, and the Biotin-free Tyramide Signal Amplification System (Dako) for DLK1. The results were analysed by three hepatologists with experience in tissue pathology without information of the specimens. HCC samples were considered positive for each HPC marker if more than 5% of tumour cells in a single section were stained [46, 47].

### Statistical analysis

Statistical analyses were performed using the SPSS software ver. 21 for Windows (IBM, New York, USA). The cut-off levels of each tumour marker associated with patient mortality were established using optimum stratification to determine the most significant *p* value by the log-rank χ^2^ statistic. As a result, we used the following cut-off values: AFP = 60 mg/dl, AFP-L3 = 20%, and DCP = 300 mAU/ml to stratify patients according to low and high serum level of each tumour marker. OS was defined as the interval between surgery and death or the last follow-up, and RFS was defined as the interval between surgery and diagnosis of any type of relapse (intrahepatic recurrence and extrahepatic metastasis were used as the end points for RFS). Univariate and multivariate analyses were based on the Cox proportional hazards regression model. OS and RFS were calculated by the Kaplan-Meier method and analysed by the log-rank test. ROC curve analysis and plotting of tumour marker serum levels were performed using Prism 6 for Windows (ver. 6.07). The *χ*^2^ test and Fisher exact probability test were used for comparison between groups. A *p* value < 0.05 was considered statistically significant.

## Abbreviations

HCC: hepatocellular carcinoma
HBV: hepatitis B virus
HCV: hepatitis C virus
NASH: non-alcoholic steatohepatitis
HPC: hepatic progenitor cell
EpCAM: epithelial cell adhesion molecule
NCAM: neural cell adhesion molecule
DLK1: delta-like 1 homolog
CK19: cytokeratin 19
AFP: α-fetoprotein
AFP-L3: lectin-reactive α-fetoprotein
DCP: des-γ-carboxy prothrombin
C-P: Child-Pugh
RFS: relapse-free survival
OS: overall survival
ROC: receiver operating characteristic
AUC: area under the curve
